# Molecular Components of the *Neurospora crassa* pH Signaling Pathway and Their Regulation by pH and the PAC-3 Transcription Factor

**DOI:** 10.1371/journal.pone.0161659

**Published:** 2016-08-24

**Authors:** Stela Virgilio, Fernanda Barbosa Cupertino, Natália Elisa Bernardes, Fernanda Zanolli Freitas, Agnes Alessandra Sekijima Takeda, Marcos Roberto de Mattos Fontes, Maria Célia Bertolini

**Affiliations:** 1 Departamento de Bioquímica e Tecnologia Química, Instituto de Química, Universidade Estadual Paulista, UNESP, 14.800-060, Araraquara, São Paulo, Brazil; 2 Departamento de Física e Biofísica, Instituto de Biociências, Universidade Estadual Paulista, UNESP, 18.618-970, Botucatu, São Paulo, Brazil; Oregon State University, UNITED STATES

## Abstract

Environmental pH induces a stress response triggering a signaling pathway whose components have been identified and characterized in several fungi. *Neurospora crassa* shares all six components of the *Aspergillus nidulans* pH signaling pathway, and we investigate here their regulation during an alkaline pH stress response. We show that the *N*. *crassa pal* mutant strains, with the exception of Δ*pal-9*, which is the *A*. *nidulans palI* homolog, exhibit low conidiation and are unable to grow at alkaline pH. Moreover, they accumulate the pigment melanin, most likely via regulation of the tyrosinase gene by the pH signaling components. The PAC-3 transcription factor binds to the tyrosinase promoter and negatively regulates its gene expression. PAC-3 also binds to all *pal* gene promoters, regulating their expression at normal growth pH and/or alkaline pH, which indicates a feedback regulation of PAC-3 in the *pal* gene expression. In addition, PAC-3 binds to the *pac-3* promoter only at alkaline pH, most likely influencing the *pac-3* expression at this pH suggesting that the activation of PAC-3 in *N*. *crassa* results from proteolytic processing and gene expression regulation by the pH signaling components. In *N*. *crassa*, PAC-3 is proteolytically processed in a single cleavage step predominately at alkaline pH; however, low levels of the processed protein can be observed at normal growth pH. We also demonstrate that PAC-3 preferentially localizes in the nucleus at alkaline pH stress and that the translocation may require the *N*. *crassa* importin-α since the PAC-3 nuclear localization signal (NLS) has a strong *in vitro* affinity with importin-α. The data presented here show that the pH signaling pathway in *N*. *crassa* shares all the components with the *A*. *nidulans* and *S*. *cerevisiae* pathways; however, it exhibits some properties not previously described in either organism.

## Introduction

All organisms adapt and survive under different environmental conditions using cellular mechanisms that integrate environmental sensing and signal transduction pathways. Extracellular pH is an environmental condition to which microorganisms need to adapt, and the signal transduction pathway mediating this adaptation has been extensively characterized in model organisms, such as the filamentous fungi *Aspergillus nidulans* and the yeast *Saccharomyces cerevisiae*. More recently, additional contributions to the role of the pH signaling pathway have been made from studies on *Candida albicans*, *Aspergillus fumigatus*, *Cryptococcus neoformans*, *Yarrowia lipolytica* and also plant pathogens, such as *Ustilago maydis*. As a consequence, their similarities and divergences and the cellular processes influenced by this signaling pathway in different organisms have been described [1, 2]. The involvement of the pH signaling pathway and the influence of the pH response for pathogenesis and virulence in pathogenic fungi have also been described, and this was first reported in *C*. *albicans* [3]. Additional studies have established that this signaling pathway plays an important role in the virulence of organisms such as *A*. *fumigatus* [4] and *C*. *neoformans* [5].

The PacC/Rim101 transcription factor in *A*. *nidulans* [6] and in *S*. *cerevisiae* [7], respectively, is the major effector that mediates the pH response. Upon a neutral to alkaline pH transition, a signaling pathway is activated leading to the activation of this transcription factor by proteolysis (reviewed in [1, 8, 9]). The Pal/Rim protein components of the pH signaling pathway are conserved among different fungal species, and three components are involved in the ambient pH sensing, the PalI/Rim9, PalH/Rim21 and PalF/Rim8 proteins in *A*. *nidulans* and *S*. *cerevisiae*, respectively. In *A*. *nidulans*, the activation of the PacC^72^ protein precursor occurs by two successive proteolytic cleavage steps [10], resulting in the active PacC^27^ protein, which translocates to the nucleus to activate alkaline-regulated genes and to repress acid-regulated genes [11]. The first step is pH dependent and is activated by the products of the six *pal* genes, while the second is proteasome-mediated and pH independent [10, 12]. Alkaline pH triggers the pH signaling pathway at cortical structures in the plasma membrane by recruiting all Pal proteins and several ESCRT (endosomal sorting complex required for transport) proteins, leading to the activation of PacC by proteolysis [1, 13–15] in a process that does not involve endocytosis, as has been recently demonstrated [16].

Although the major components of the pH signaling pathway are conserved among different organisms, there are differences between the PacC and Rim101 pathways, and one major difference is that the Rim101 transcription factor requires only a single proteolytic cleavage step to be activated in *S*. *cerevisiae* [17] and *C*. *albicans* [18]. Additionally, in *A*. *nidulans* and *S*. *cerevisiae*, PalF/Rim8, respectively, are post-translationally modified by ubiquitination, while *C*. *albicans* Rim8 is phosphorylated in response to a neutral-alkaline pH transition, and this modification correlates with Rim101 activation [19]. In *A*. *nidulans*, ubiquitination of PalF plays a key role in pH signaling, promoting the downstream events of the pathway [20]. More recently, new components in the pH response pathway have been described. Thus, in *A*. *nidulans*, the zinc binuclear DNA binding protein PacX was identified and characterized, and the authors suggested that PacX plays a role in *pacC* gene repression and in PacC^27^ activity [21]. Interestingly, this protein is absent in Saccharomycotina [21]. In *C*. *neoformans*, the RRA1 protein, which has a predicted structure similar to PalH/Rim21, was shown to be required for Rim101 activation [22].

In *Neurospora crassa*, the influence of the pH signaling pathway in metabolism was first described by demonstrating that the PACC transcription factor affects glycogen levels, more likely by controlling the expression of the gene encoding glycogen synthase (*gsn*), the regulatory enzyme involved in glycogen synthesis [23]. The PACC pathway was reported to be involved in glycosylation of Pi-repressible acid phosphatase [24], the transcription of the *hsp70* gene [25] and the requirement for female development [26] in *N*. *crassa*. Although this signaling pathway influences several cellular processes in *N*. *crassa*, no studies have evaluated the components of this signaling pathway in the pH response. In this work, we describe the characterization of the protein components of this signaling pathway in *N*. *crassa*, and we demonstrate that all mutant strains, with the exception of Δ*pal-9*, overproduce melanin, most likely due to high expression of the tyrosinase gene in these mutant strains. We further describe the modulation of the expression of the *pal* genes by alkaline pH and by the PAC-3 transcription factor and demonstrate the existence of feedback regulation involving PAC-3 at alkaline pH. Finally, we show that PAC-3 migrated to the nucleus at alkaline pH and that this translocation may occur by the classical nuclear import pathway [27] through the interaction between the *N*. *crassa* importin-α and a specific basic region of PAC-3, which has a nuclear localization signal (NLS).

## Materials and Methods

### *Neurospora crassa* strains and culture conditions

*Neurospora crassa* FGSC#9718 (*mat a*, *mus-51*::*bar*), the wild-type background strain, and the FGSC#21931 (Δ*pal-1*, *mat a*), FGSC#15867 (Δ*pal-2*, *mat A*), FGSC#16419 (Δ*pal-3*, *mat a*), FGSC#22412 (Δ*pal-6*, *mat a*), FGSC#16099 (Δ*pal-8*, *mat a*) and FGSC#13378 (Δ*pal-9*, *mat a*) mutant strains were purchased from the Fungal Genetics Stock Center (FGSC, University of Missouri, Kansas City, MO, USA, http://www.fgsc.net) [28]. The Δ*pac-3* strain was generated as described in Cupertino et al. [23]. Details for all mutant strains can be found in S1 Table. The gene knockout in all mutant strains was confirmed by PCR using specific oligonucleotides (S2 Table, real-time PCR primers) by comparing to the amplification of genomic DNA from the wild-type strain. The strains were maintained on solid Vogel’s minimal (VM) medium, pH 5.8 [29] containing 2% sucrose at 30°C. Conidia from 10-day old cultures of wild-type and mutant strains were suspended in sterile water and counted. For morphology analyses, 10^7^ conidia/mL were inoculated into flasks containing solid VM medium plus 2% sucrose, pH 5.8. For radial growth analyses, 10^7^ conidia/mL were inoculated onto Petri dishes containing solid VM medium plus 2% sucrose at pH 5.8 and 7.8 for 24 h at 30°C. Images of colony morphology were captured after 24 h.

For pH stress, 10^9^ conidia/mL were first germinated in 1 L of VM medium, pH 5.8, at 30°C and 200 rpm for 24 h. After this period, the culture was filtered and the mycelia were divided into two samples. One was frozen in liquid nitrogen and stored at -80°C for further processing (control sample, not submitted to stress), while the remaining sample was transferred to 500 mL of fresh VM medium containing 0.5% sucrose at pH 7.8 (for alkaline pH stress). Sample from mycelia submitted to pH stress was harvested after 1 h incubation. The mycelial samples were used for RNA extraction and gene expression assays.

### Construction of the Δ*pac-3* complemented strain

For complementation, the strain Δ*pac-3*::*bar* [23] was crossed with the *his-3* mutant strain (FGSC#6103, *A his-3*) to generate the Δ*pac-3 his-3* double mutant strain. A DNA fragment of 2,041 bp was amplified by PCR with the primers N-mChPACC-F and N-mChPACC-R (S2 Table) using genomic DNA from the wild-type strain as a template. The N-mChPACC-F oligonucleotide contains the sequence that codifies for 6-Gly between the nucleotide sequences encoding mCherry and the PAC-3 protein. PCR was performed using a Phusion High-Fidelity PCR kit (Finzymes), and the DNA fragment was purified with a QIAquick Gel Extraction Kit (Qiagen, CA) according to the manufacturer’s instructions. The purified DNA fragment was cloned into the *Spe*I and *Xba*I sites of the pTSL48-B plasmid (a donation from N. L. Glass, University of California at Berkeley, Berkeley, CA, USA), generating the pTSL48-B-*pac-3* plasmid. The pTSL48-B plasmid allows the constitutive expression of the N-terminus mCh-PAC-3 fusion protein, as the *ccg-1* promoter drives the *pac-3* gene expression. The pTSL48-B-*pac-3* construction was used to transform competent conidia from the recipient Δ*pac-3 his-3* double mutant strain. The transformants (Δ*pac-3 his-3*::*ccg-1-mCh-pac-3*) were selected on VM media containing basta without histidine and confirmed by PCR using the primers N-mChPACC-F and N-mChPACC-R (S2 Table). The progeny were analyzed by fluorescence microscopy and by evaluating the growth on Petri dishes and tubes for the production of melanin. The complemented strain (Δ*pac-3 pac-3*^+^) was evaluated by growing on solid VM medium containing 2% sucrose at pH 5.8 and 7.8 and comparing to the wild-type and *pac-3* mutant strains.

### RNA extraction and gene expression assays

For the RT-qPCR analysis, total RNA from the wild-type strain and the Δ*pac-3*, Δ*pal-1*, Δ*pal-2*, Δ*pal-3*, Δ*pal-6*, Δ*pal-8* and Δ*pal-9* mutants was prepared using mycelia samples cultured at pH 5.8 for 24 h at 30°C and mycelia grown at pH 5.8 and shifted to pH 7.8 for 1 h, according to Sokolovsky et al. [30]. Expression of the tyrosinase (NCU00776) gene and the *pal-1* (NCU05876), *pal-2* (NCU00317), *pal-3* (NCU03316), *pal-6* (NCU03021), *pal-8* (NCU00007), *pal-9* (NCU01996) and the *pac-3* (NCU00090) genes was evaluated by RT-qPCR using specific oligonucleotides (S2 Table). For this, total RNA (10 μg) samples were first treated with RQ1 RNase-free DNase (Promega) and subjected to cDNA synthesis by using SuperScript III First-Strand Synthesis kit (Invitrogen) and an oligo (dT) primer, according to manufacturer’s instructions. The cDNA libraries were subjected to RT-qPCR on a StepOnePlus^™^ Real Time PCR System (Applied Biosystems) using the Power SYBR^®^ Green PCR Master Mix (Applied Biosystems) and specific primers for each gene amplicon (S2 Table). Reactions were performed under the following conditions: 95°C for 10 min, 40 cycles of 95°C for 15 s, 60°C for 1 min to calculate cycle threshold (Ct) values, followed by 95°C for 15 s, 60°C for 1 min and then 95°C for 15 s to obtain melt curves. Data analysis was performed by the StepOne Software (Applied Biosystems) using the comparative CT (ΔΔCT) method [31]. At least three biological replicates, with three experimental replicates per sample were performed, and reactions with no template were used as a negative control. The fluorescent dye ROX^™^ was used as the passive reference to normalize the SYBR green reporter dye fluorescence signal. The PCR products were subjected to melting curves analysis to verify the presence of a single amplicon. All reaction efficiencies varied from 94 to 100%. We used the expression of the beta-tubulin gene as the reference gene (β-*tub-2* gene, NCU04054).

### Protein expression

Conidia (10^8^/mL) from the Δ*pac-3* complemented and wild-type strains were grown in 500 mL of VM liquid medium containing 2% sucrose, pH 5.8 at 30°C and 200 rpm for 24 h. After that, the culture was filtered, and the mycelia were divided into three samples. The control sample was not subjected to pH stress and was frozen in liquid nitrogen and stored at -80°C. The remaining samples were transferred into 250 mL of fresh VM medium containing 0.5% sucrose either at pH 4.2 (for acid stress) or 7.8 (for alkaline stress). Samples were harvested after 1 h incubation at 30°C and 200 rpm and frozen at -80°C. Mycelia pads were disrupted by grinding in a mortar with liquid nitrogen, and proteins were extracted with extraction buffer (50 mM HEPES, pH 7.4, 137 mM NaCl, 10% glycerol, 1 mM PMSF, 0.1 mM TCLK, 1 mM benzamidine, and 1 μg/ml of each pepstatin and antipain) [32] plus 200 μL of glass beads (710–1180 μm, Sigma) and quantified by the Hartree method [33] using BSA as standard. The amounts of 50 and 70 μg of total protein were separated by 10% SDS-PAGE gels [34] and electro-transferred to nitrocellulose blotting membrane (GE Healthcare). Immunoblotting was performed with a polyclonal anti-mCherry antibody (BioVision). Blots were subsequently probed with HRP-conjugated secondary antibodies (Sigma) and developed with luminol reagent.

### Chromatin immunoprecipitation-PCR assays

Chromatin immunoprecipitation assays were performed as described by Tamaru et al. [35], with modifications. Briefly, conidia from the Δ*pac-3* complemented strain were grown in 250 mL of VM liquid medium containing 2% sucrose pH 5.8 at 30°C and 200 rpm for 24 h. After that, mycelium from half of the culture was collected by filtration and transferred into 125 mL of fresh VM medium containing 0.5% sucrose pH 7.8 and incubated at 30°C and 200 rpm for 1 h. The remaining sample (control, not subjected to pH stress) and the sample subjected to pH stress were fixed by adding formaldehyde (Sigma) to final concentration of 1%, followed by incubation for 30 min at 30°C and 200 rpm. Formaldehyde was quenched using 125 mM glycine at 30°C and 200 rpm during 10 min. Both samples were subsequently harvested by filtration and suspended in ChIP lysis buffer (50 mM HEPES, pH 7.9, 90 mM NaCl, 1 mM EDTA pH 8.0, 1% Triton X-100, 0.1% sodium deoxycholate, 1 mM PMSF, 0.1 mM TCLK, 1 mM benzamidine, and 1 μg/ml of each pepstatin and antipain). The chromatin was sheared to an average size of 0.3–0.8 kb using Vibra Cell Sonics ultrasonic processor (10 cycles: 1 min, 40% amplitude, 8.0 s pulse ON, 9.9 s pulse OFF on ice). Extracts were clarified by centrifugation, and the sonicated chromatin was pre-cleared with Dynabeads Protein A (Novex) pre-blocked with 0.5% BSA in PBS 1x and then immunoprecipitated with the anti-mCherry polyclonal antibody (BioVision) and Dynabeads Protein A. The DNA was quantified using NanoVue Plus spectrophotometer (GE Healthcare), and 25 ng of input DNA (I), no Ab (N, reaction without antibody) and IP (immunoprecipitated DNAs with anti-mCherry antibody) samples were amplified by PCR using primers specific for each promoter (S2 Table). Input DNA was used as a positive control of the experiment, and no Ab was used as a negative control.

PCR was performed using a Phusion High-Fidelity PCR kit (Finzymes) and specific oligonucleotides (S2 Table) for the tyrosinase (tyrp-F/tyrp-R), *pal-1* (pal1p-F/pal1p-R), *pal-2* (pal2p-F/pal2p-R), *pal-3* (pal3p-F/pal3p-R), *pal-6* (pal6p-F/pal6p-R), *pal-8* (pal8p-F/pal8p-R), *pal-9* (pal9p-F/pal9p-R) and *pac-3* (pac3p-F/pac3p-R) promoters. An ubiquitin gene fragment (NCU05995) amplified by the primers qUbi-F/qUbi-R was used as a negative control for binding. The ubiquitin fragment does not have the PAC-3 motif. Reactions were performed under the following conditions: 98°C for 10 s, 25 cycles of 98°C for 1 s, 60°C for 5 s and 72°C for 30 s, and then 72°C for 5 min. The reaction products were analyzed on a 2% agarose gel and visualized by ethidium bromide. Densitometry was performed using ImageJ software [36], and the IP signals were compared to the negative control (no Ab).

### Subcellular localization

To determine the subcellular localization of the fluorescent mCh-PAC-3 protein at different pH conditions, 40 μL of a 3 x 10^8^ conidia/mL suspension from the Δ*pac-3* complemented strain was inoculated onto coverslips, covered with VM liquid plus 1% sucrose, pH 5.8 and incubated at 30°C for 12 h. After this period, the coverslips were transferred to fresh VM media containing 1% sucrose, pH 7.8 at 30°C and incubated for 30 min and 1 h. For nuclei analysis, mycelia were fixed [1% phosphate buffered saline (PBS), 3.7% formaldehyde, 5% DMSO], washed twice with PBS and stained with 100 μl DAPI (4',6-diamidino-2-phenylindole, 0.5 mg/mL) for 5 min. DAPI fluorescence was visualized using a fluorescence microscope with excitation and emission wavelengths of 358 nm and 463 nm, respectively, and mCherry fluorescence was visualized using excitation and emission wavelengths of 563 nm and 581 nm, respectively. The images were captured using an AXIO Imager.A2 Zeiss microscope, at a magnification of 100 X, coupled to an AxioCam MRm camera and processed using the AxioVision software, version 4.8.2. Further processing was performed using Corel^®^PHOTO-PAINT^™^ X7.

### Expression and purification of importin-α and synthesis of PAC-3 NLS

The gene (NCU01249) encoding importin-α was cloned into the pET28a expression vector, and the recombinant protein was expressed as a truncated protein consisting of residues 75–529 fused to a 6-His tag at N-terminus, as previously described [37]. The protein was expressed in the *Escherichia coli* host strain RosettaTM (DE3) pLysS (Novagen) and purified by affinity chromatography [37]. The protein was eluted with a 0.15–3.0 M imidazole linear gradient, followed by dialysis in buffer (20 mM Tris-HCl, pH 8.0 and 100 mM NaCl) and stored at cryogenic temperatures. The PAC-3 NLS peptide (281FDARKRQFDDLNDFFGSVKRRQIN304) used in the experiments was synthesized with purity higher than 95% (GenOne). The peptide contained additional residues at the N- and C-termini compared with the minimally identified NLS to avoid artifactual binding at the termini [38].

### Isothermal titration calorimetry (ITC)

ITC experiments were carried out to verify the binding affinity of importin-α to the putative NLS peptide of PAC-3 (PAC-3 NLS). The experiments were performed with a MicroCal iTC200 microcalorimeter (GE Healthcare). The protein and the PAC-3 NLS peptide were diluted in buffer (20 mM Tris-HCl, pH 8.0 and 100 mM NaCl) at a concentration of 50 μM and 500 μM for protein and peptide (protein:peptide molar ratio of 1:10), respectively. The protein sample was added to the cell, and the peptide was titrated into the cell with a syringe. Titrations were conducted at 20°C and consisted of 20 injections of 2.0 μL in an interval of 240 s with an 800 rpm homogenization speed. The heat of the dilution was determined in a control assay by titration of the peptide sample into the protein sample buffer and was subtracted from the corresponding titrations. The data were processed using MicroCal Origin Software to obtain values for stoichiometry (N), dissociation constants (K_d_), and enthalpy (ΔH). The binding-type input parameters were adjusted to obtain the best fitting model. The values of K_d_ and ΔH were used to calculate free energy (ΔG) and entropy (ΔS) values.

## Results

### The Δ*pal* and Δ*pac-3* mutant strains show impaired growth at alkaline pH and high production of melanin

In *A*. *nidulans*, the pH signaling pathway includes the central regulator PacC, which undergoes proteolytic processing at a neutral to alkaline pH transition in a process mediated by the pH-dependent *pal* gene cascade and the proteasome [11]. *N*. *crassa* has the six *A*. *nidulans pal* gene homologs, and to characterize the putative *N*. *crassa* PAL proteins, we first analyzed the morphology of the Δ*pal* mutant strains. The gene knockout in all strains was confirmed by PCR using the oligonucleotides described in S2 Table (Real-time PCR primers). All *N*. *crassa pal* and *pacC* mutant strains were renamed here considering the Neurospora nomenclature [39], replacing the letters on the *A*. *nidulans* gene names by numbers (S1 Table, last column). S1 Table also shows the FGSC number and the mating type of the mutant strains, the ORF number, the theoretical MW and pI, the protein family or domain of the PAL and PAC-3 proteins and the annotation of ortholog proteins in fungi and yeast.

**Fig 1:**
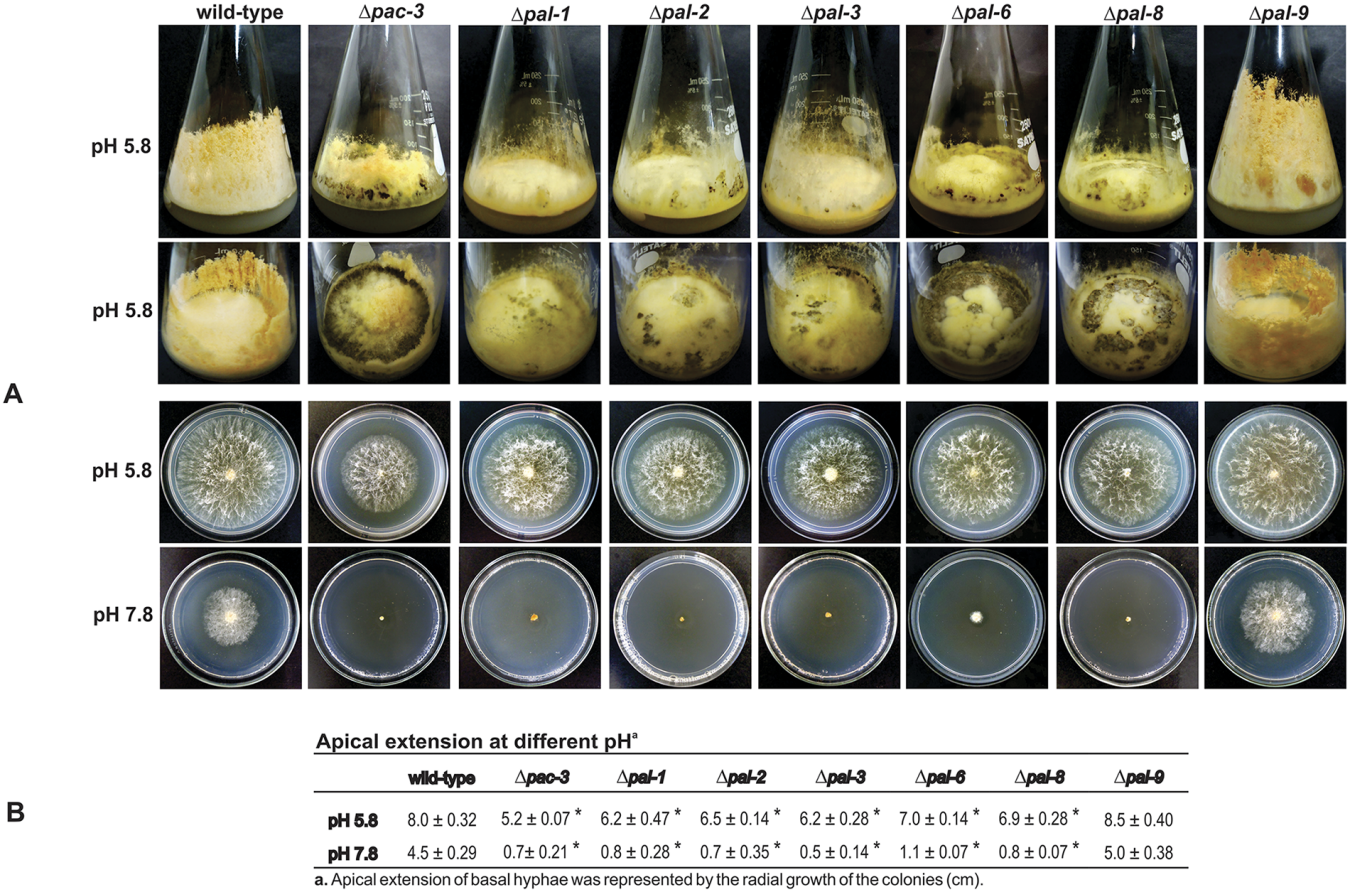
Morphological analyses of the *pal* mutant strains. **(A)** The strains (10^7^ conidia/mL) were cultured in Erlenmeyer flasks containing solid VM medium plus 2% sucrose at pH 5.8 for 8–10 days. Basal hyphae growth was examined after cultivating the strains on Petri dishes containing solid VM medium plus 2% sucrose at pH 5.8 and 7.8 for 24 h at 30°C. **(B)** Radial growth of the colonies measured in cm. The results represent at least two independent experiments. *Indicates significant difference between wild-type and mutant strains at the same pH (Student’s *t*-test, *P* < 0.01).

**Fig 2:**
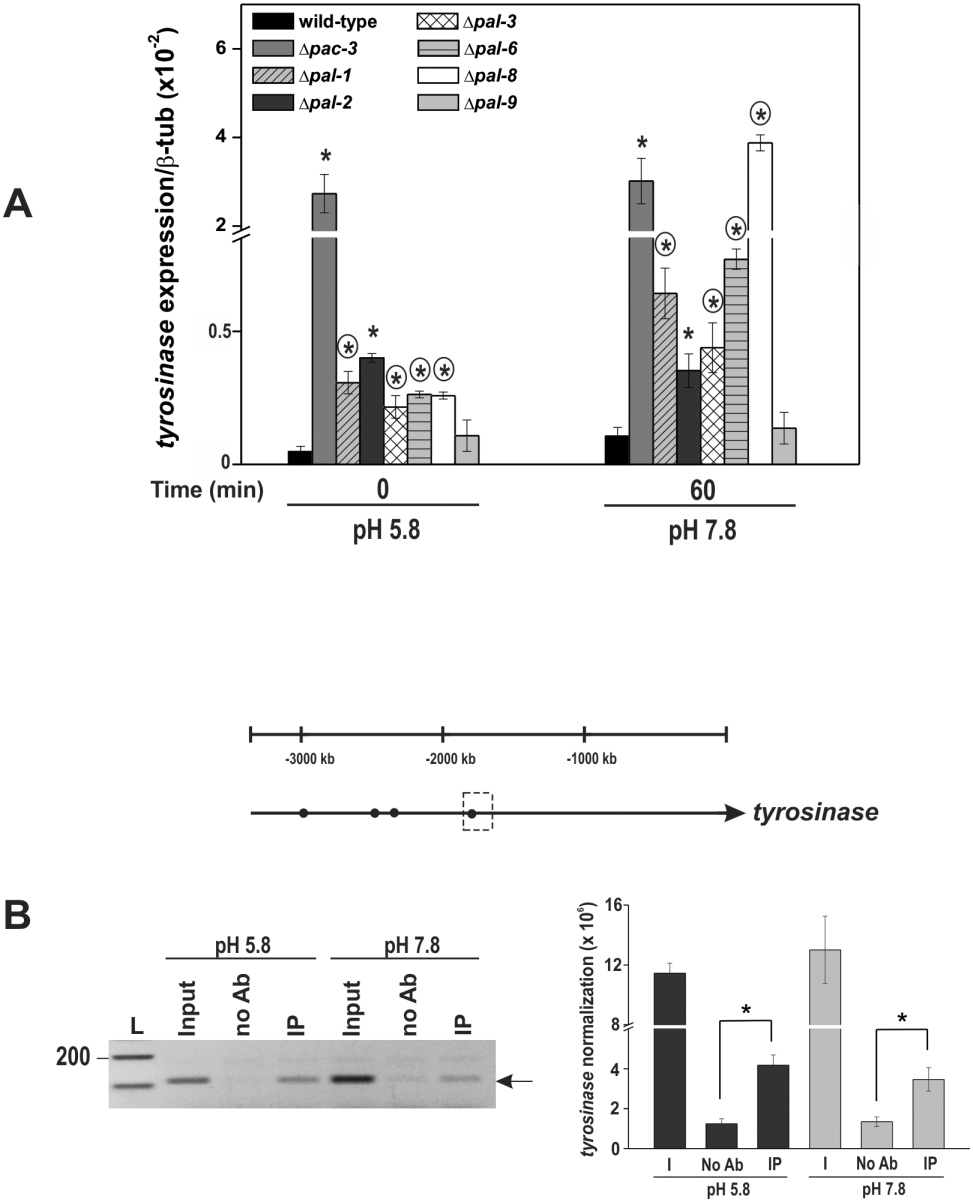
Expression of the tyrosinase gene in the wild-type and the Δ*pal* mutant strains at normal (5.8) and alkaline (7.8) pH. **(A)** Mycelial samples from the wild-type and Δ*pal* mutant strains cultured at pH 5.8 for 24 h and shifted to pH 7.8 for 1 h were used to extract total RNA. Gene expression analysis was performed by RT-qPCR on the StepOnePlus^™^ Real-Time PCR system (Applied Biosystems) using Power SYBR^®^ Green and specific primers. The *tub-2* gene was used as the reference gene. The asterisks indicate significant differences compared to the wild-type strain at the same pH, and circles indicate significant differences between the same mutant strain cultured at a different pH (Student’s *t*-test, *P* < 0.01). **(B)** Representation of the PAC-3 motifs (black circles) in the tyrosinase gene promoter. Dashed boxes indicate the region analyzed by ChIP-PCR. Genomic DNA samples from the Δ*pac-3* complemented strain subjected to pH stress or not were immunoprecipitated with an anti-mCherry antibody and subjected to PCR using specific primers. The input DNA (I) was used as the positive control, and the reactions without any antibody (no Ab) were used as the negative control. The intensity of the DNA bands in the gel (left side, arrow) was quantified by ImageJ and the results are shown in the right figure. L, 1 kb DNA ladder. *Asterisks indicate significant differences between the no Ab and IP samples at the same pH (Student’s *t*-test, *P* < 0.01). All results represent the average of at least three independent experiments. Bars indicate the standard deviation from the biological experiments.

To assess the effects of the knockout genes, we analyzed the Δ*pal*-1, Δ*pal-2*, Δ*pal-3*, Δ*pal-6*, Δ*pal-8*, Δ*pal-9* and Δ*pac-3* strains morphology at normal (5.8) and alkaline (7.8) pH. Ten-day old flask cultures at pH 5.8 exhibited reduced aerial growth, low conidiation and high production of a dark pigment, except Δ*pal-9* (the *palI* homolog), compared to the wild-type strain (Fig 1A). The Δ*pac-3* (the *pacC* homolog) and Δ*pal-6* (the *palF* homolog) strains showed the highest pigmentation among all the Δ*pal* mutants. Basal hyphae growth was examined after cultivating the strains on Petri dishes at pH 5.8 and 7.8 for 24 h. All mutant strains, except Δ*pal-9* (the *palI* homolog), were able to growth at pH 5.8; however, they were unable to grow at alkaline pH (7.8) (Fig 1A). Notably, all mutant strains showed reduced radial growth compared to the wild-type strain. The apical extension of the colonies at different pH values was measured and is presented in Fig 1B. These results suggest that PAC-3 and the PAL proteins, except PAL-9 (the PalI homolog), are required for growth under alkaline conditions, confirming that the *N*. *crassa pal* genes are involved in the response to alkaline pH stress as in *A*. *nidulans*. As most of the mutant strains showed high production of a dark pigment, likely melanin, we analyzed the levels of the tyrosinase gene (NCU00776) in the strains at pH 5.8 and 7.8 by RT-qPCR. Tyrosinase is a rate-limiting enzyme that controls the production of melanin [40]. The β-tubulin gene (*tub-2*, NCU04054) was used as the endogenous control. The tyrosinase gene was expressed in all mutant strains at normal growth pH (5.8) and over-expressed at alkaline pH (*P* < 0.01), except in Δ*pal-9*, which did not produce the dark pigment. These results indicate that the expression of the gene was also regulated by alkaline pH (Fig 2A) and that the PAC-3 and PAL proteins may control the melanin biosynthesis by negatively regulating the tyrosinase expression. It is important to observe the high tyrosinase gene expression in the Δ*pac-3* strain, indicating that PAC-3, the final component of the pH signaling pathway, plays a key regulatory role in the tyrosinase expression.

An *in silico* analysis of the tyrosinase gene promoter revealed the existence of four *N*. *crassa* PAC-3 DNA binding preference (5’-BGCCVAGV-3’) [41] in its 5’-flanking region (Fig 2B). ChIP-PCR assays were performed to confirm the regulation of the tyrosinase gene by PAC-3. One PAC-3 motif was analyzed for *in vivo* binding (the dashed box in Fig 2B). In these experiments, we used the Δ*pac-3* complemented strain (Δ*pac-3 his-3*::*Pccg-1-mCh-pac-3*) and an anti-mCherry antibody. Chromatin was collected from the mycelial samples grown for 24 h at pH 5.8 for 1 h after transfer to pH 7.8. The input DNA (I) and the reactions without antibody (no Ab) were used as positive and negative controls of the experiment, respectively. The results showed that PAC-3 bound to the tyrosinase promoter *in vivo*, under normal and alkaline pH conditions (Fig 2B). This DNA-protein binding may explain the high expression of the tyrosinase gene and most likely the high melanin biosynthesis by the mutant strains.

### *pac-3* complementation rescues the wild-type phenotype

To confirm that the morphological changes in the Δ*pac-3* (the *pacC* homolog) strain were due to the *pac-3* knockout, we constructed the Δ*pac-3* complemented strain (Δ*pac-3 his-3*::*Pccg-1-mCh-pac-3*) by inserting the *pac-3* genomic sequence from the wild-type strain into the *his-3* locus. When cultured under the same conditions as the mutant and the wild-type strains, the complemented strain (Δ*pac-3 pac-3*^+^) restored the wild-type phenotype, confirming that the morphological aspects observed in the mutant strain are indeed due to the *pac-3* deletion (S1 Fig). We first analyzed growth of the Δ*pac-3* and the Δ*pac-3* complemented strains in tubes of 10-day cultures at pH 5.8. The complemented strain did not show melanin pigmentation and exhibited a similar phenotype to the wild-type strain (S1A Fig). To investigate the pH response in the complemented strain, we evaluated its sensitivity to pH stress in cultures after 24 h of growth at 30°C and compared the results to the Δ*pac-3* and wild-type strains. The Δ*pac-3* mutant strain was sensitive to both pH analyzed, exhibiting lower radial growth than the wild-type strain at pH 5.8 and an inability to grow at alkaline pH (7.8), while the rescued strain exhibited a wild-type phenotype at both pH values (S1B Fig). The results confirm that the effects of pH on the growth sensitivity and melanin production were indeed due to the lack of the active PAC-3 transcription factor.

### PAC-3 processing is pH-dependent

**Fig 3:**
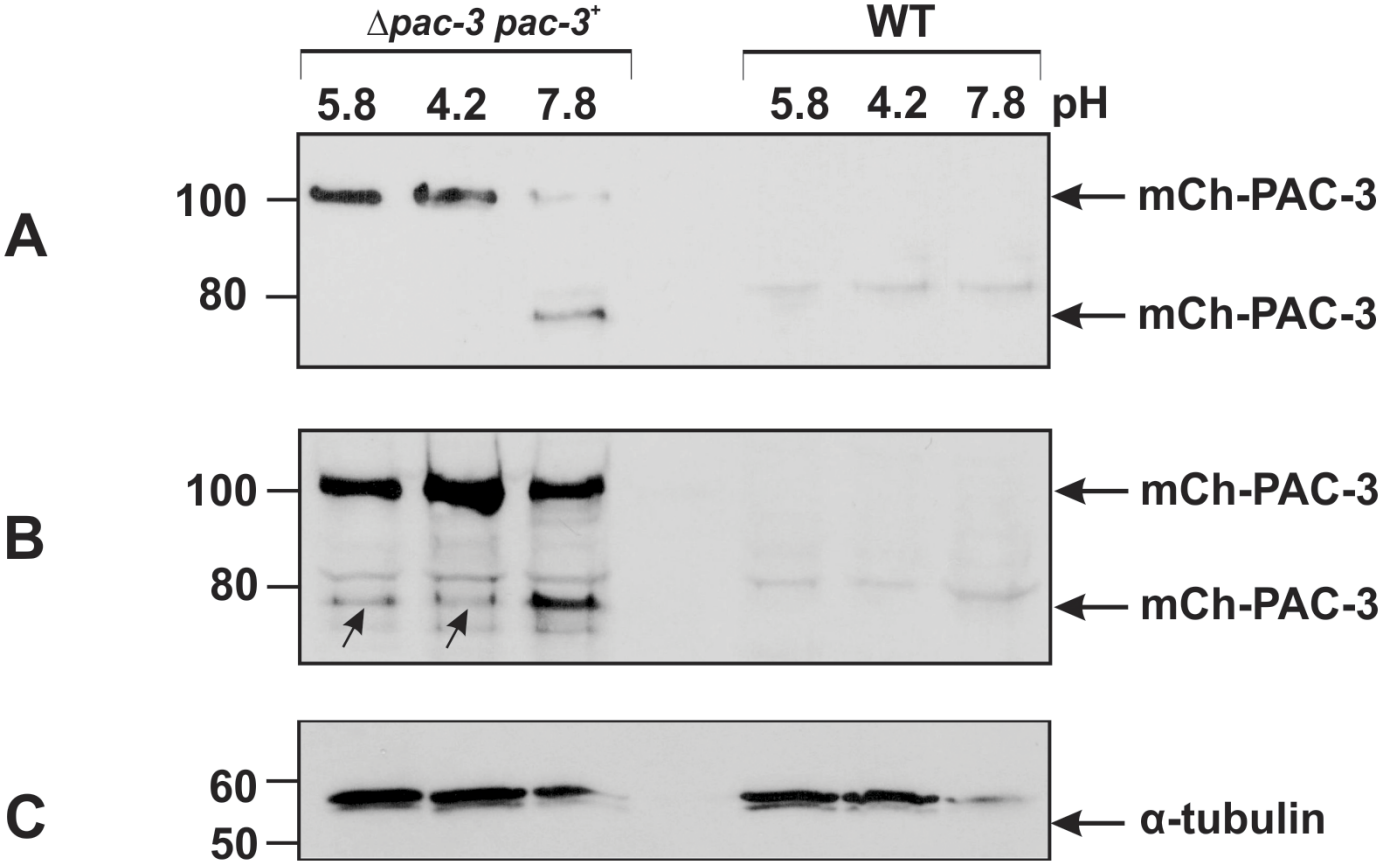
PAC-3 proteolytic processing at alkaline pH. PAC-3 protein levels were detected by western blot using the polyclonal anti-mCherry antibody and cell extracts from the wild-type and complemented (Δ*pac-3 pac-3*^+^) strains cultivated at pH 5.8 at 30°C for 24 h and then shifted to pH 4.2 and 7.8 at 30°C for 1 h. **(A)** Aliquots of 50 μg of total protein were loaded onto the gel. **(B)** Aliquots of 70 μg of total protein were loaded onto the gel. The arrows indicate the processed mCh-PAC-3 form at pH 5.8 and 4.2. **(C)** The protein α-tubulin was used as the loading control. The plots represent one of the three independent experiments. The numbers on the left represent the molecular weight in kD.

The *A*. *nidulans* PacC undergoes two proteolytic cleavage steps, while the *S*. *cerevisiae* Rim101p undergoes only one cleavage step for activation after transfer to alkaline pH. Therefore, we investigated whether PAC-3 undergoes proteolytic cleavages, resulting in isoforms with different molecular masses, and we used the Δ*pac-3* complemented strain in this experiment. The presence of PAC-3 was analyzed in cellular extracts from mycelia from wild-type and complemented strains cultivated at pH 5.8 (not subjected to pH stress) and from mycelia collected after transferring to pH 4.2 and 7.8 (for acid and alkaline stress, respectively) for 1 h. The protein was detected using the anti-mCherry antibody. In the complemented strain, PAC-3 is fused to mCherry having a final molecular weight of approximately 100 kD, which corresponds to 67.3 kD of the full-length PAC-3 protein (621 amino acid residues) along with 28.8 kD of mCherry (237 amino acid residues). The mCh-PAC-3 protein was predominantly detected in 50 μg of total protein from crude cellular extracts prepared from cultures grown at pH 5.8 and 4.2 and was barely detected at pH 7.8 (Fig 3A). A single proteolytic cleavage was observed only at pH 7.8, leading to the production of a fused protein showing a molecular mass close to 80 kD (Fig 3A), indicating that the size of the PAC-3 processed form is similar to the intermediate PacC form from *A*. *nidulans* grown at alkaline pH (53 kD). It is important to mention that the *N*. *crassa* PAC-3 processed form was detected in cell extracts prepared from mycelium subjected to alkaline pH stress for 1 h, the time necessary for full PacC processing in *A*. *nidulans*. The second processed PacC form (PacC^27^) described in *A*. *nidulans* was not detected in *N*. *crassa*.

We also analyzed 70 μg of total protein and observed the predominant full-length form of mCh-PAC-3 and one proteolytically processed form at pH 7.8 (Fig 3B). However, the same PAC-3 processed form was also observed at lower levels at pH 5.8 and 4.2 (arrows), indicating that PAC-3 may be proteolytically processed independent of the alkaline pH signaling pathway (Fig 3B). As expected, the mCh-PAC-3 protein was not detected in the wild-type cellular extracts. The blot probed with anti-α-tubulin antibody was used as loading control (Fig 3C). Based on these results, we conclude that PAC-3 undergoes only one pH-dependent proteolytic cleavage, similar to what is described for the *S*. *cerevisiae* and *C*. *albicans* Rim101 processing at alkaline pH. Interestingly, our results also show that PAC-3 could be processed in a pH-independent manner (pH 5.8 and pH 4.2), most likely independent of the *pal* signaling cascade.

### The PAC-3 transcription factor binds to all *pal* gene promoters *in vivo* and influences their expression

**Fig 4:**
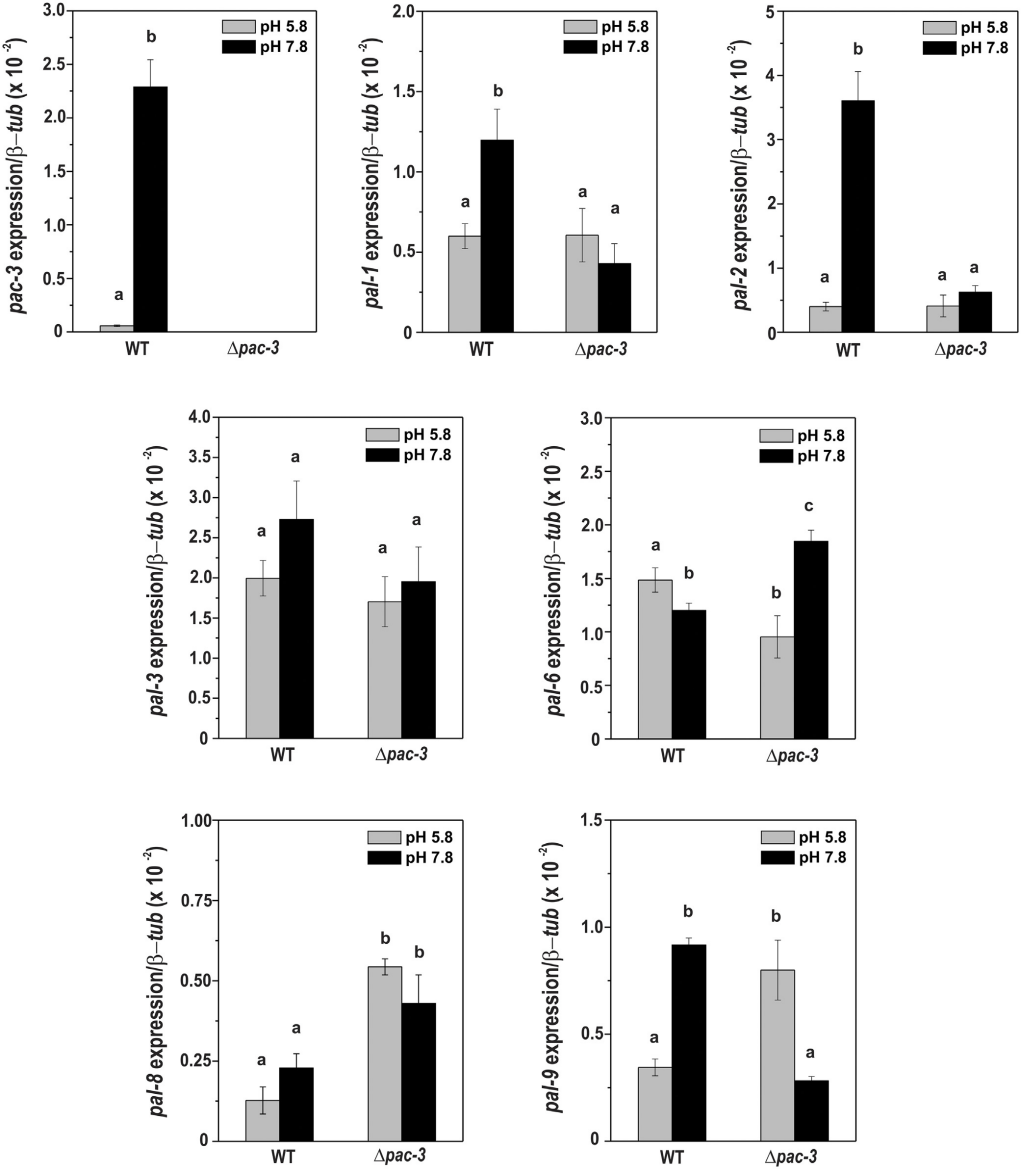
Expression of the *pal* genes in the wild-type and Δ*pac-3* strains at normal and alkaline pH. Cells from the wild-type and Δ*pac-3* strains were cultured at pH 5.8 for 24 h and shifted to pH 7.8 for 1 h. Mycelial samples were collected and used to extract total RNA. Gene expression analysis was performed by RT-qPCR in the StepOnePlus^™^ Real-Time PCR system (Applied Biosystems) using the Power SYBR^®^ Green and specific primers. The *tub-2* gene was used as the reference gene, and the pH 5.8 wild-type was used as the reference sample. At least three biological replicates were performed, and the data were analyzed using the relative quantification standard curve method. Bars indicate the standard deviation from the biological experiments. **a, b, c:** Letters above the bars indicate statistical significance; different letters indicate significant differences between two samples and similar letters indicate no significant difference between two samples at the same or different pH (Student’s *t*-test, *P* < 0.01).

To investigate whether PAC-3 feedback controls the pH signaling by regulating the *pal* and *pac-3* genes, we analyzed the expression of all genes in mycelia from Δ*pac-3* and wild-type strains grown at pH 5.8 for 24 h and in mycelia shifted to pH 7.8 for 1 h. The expression of all genes was analyzed by RT-qPCR using specific oligonucleotides described in S2 Table. The *tub-2* gene was used as the reference gene and the wild-type sample at pH 5.8 as the reference sample. Expression of the *pac-3* gene in the wild-type strain was significantly higher at alkaline pH (*P* < 0.01), as expected (Fig 4) and consistent with previous results obtained by Cupertino et al. [23]. Additionally, the *pal-1* (the *palA* homolog), *pal-2* (the *palB* homolog), and *pal-9* (the *palI* homolog) genes were over-expressed at alkaline pH in the wild-type strain, and their expression was dependent on the PAC-3 transcription factor since they were down-regulated in Δ*pac-3* strain under the same pH (Fig 4). Curiously, the Δ*pal-9* strain grew well at pH 7.8 and did produce melanin, although the *pal-9* expression was regulated by alkaline pH. On the other hand, the expression of the *pal-6* (the *palF* homolog) gene was significantly down-regulated in the wild-type strain at pH 7.8 (*P* < 0.01) and dependent on the PAC-3 transcription factor. Finally, the expression of the *pal-3* (the *palC* homolog) gene however, was not influenced by PAC-3 or pH 7.8, and while the expression of *pal-8* (the *palH* homolog, the pH sensor) gene was dependent on PAC-3, it was not influenced by pH 7.8 in the wild-type strain (Fig 4). In summary, the expression of *pal* genes was differentially regulated by alkaline pH and by the PAC-3 transcription factor.

**Fig 5:**
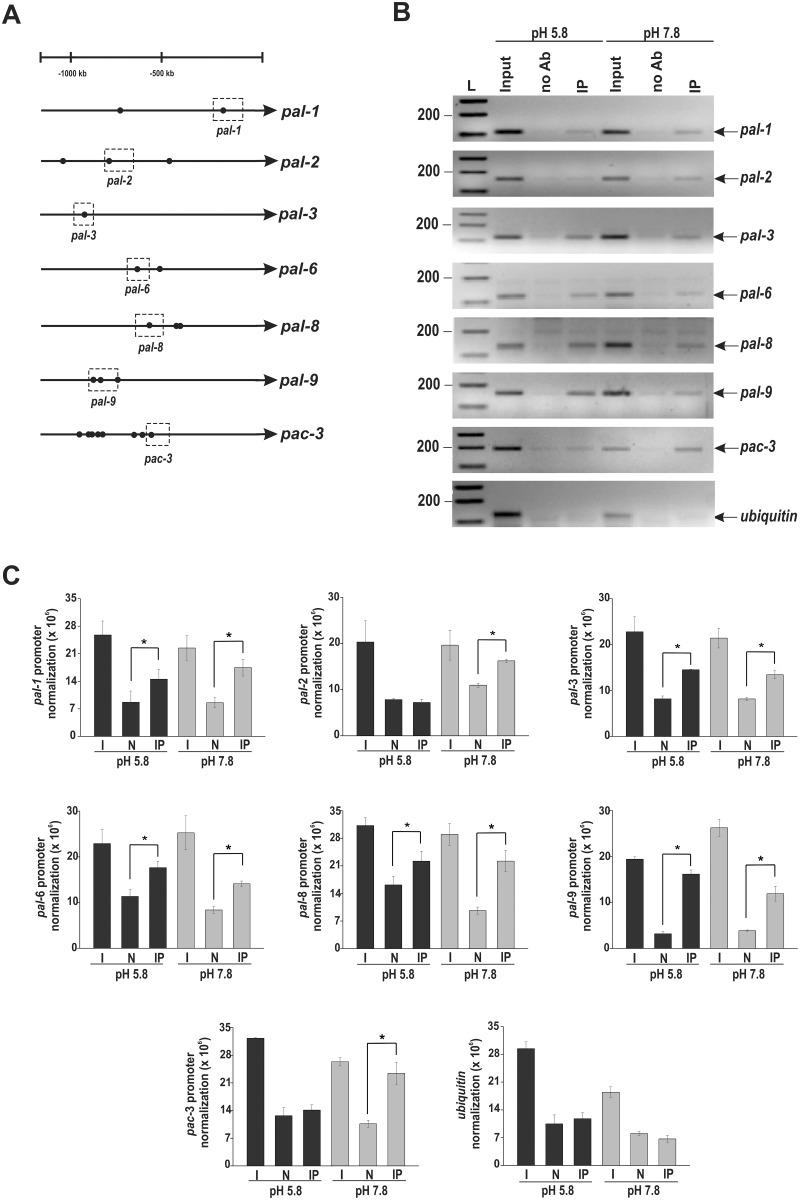
Binding of PAC-3 to the *pal* gene promoters at normal and alkaline pH. **(A)** Representation of the PAC-3 motifs in the *pal* gene promoters. The black dots indicate the position of the PAC-3 motifs, and the dashed boxes indicate regions that were analyzed by ChIP-PCR. **(B)** Genomic DNA samples from the Δ*pac-3* complemented strain both subjected to alkaline pH stress or not were immunoprecipitated with the anti-mCherry antibody and subjected to PCR to amplify DNA fragments containing the PAC-3 motif. A DNA fragment from the ubiquitin gene, which does not have a PAC-3 motif, was used as a negative control of binding. The input DNA (I) was used as a positive control and the non-immunoprecipitated reaction (no Ab) as the negative control. L, 1 kb DNA ladder. **(C)** The DNA bands after ChIP-PCR were quantified by ImageJ and the results are shown in the graphs. *Asterisks indicate significant differences between no Ab and IP at the same pH (Student’s *t*-test, *P* < 0.01). All results represent the average of at least two independent experiments. Bars indicate the standard deviation from the biological experiments.

An *in silico* analysis of the *pal* gene promoters revealed the existence of the *N*. *crassa* PAC-3 motif in all promoters, either adjacent to or distant from each other (Fig 5A), suggesting that the *N*. *crassa* PAC-3 transcription factor could directly regulate the expression of these genes. Many PAC-3 motifs were also identified within the *pac-3* gene promoter. A ChIP-PCR assay was performed to analyze whether PAC-3 could bind to DNA fragments containing some of these motifs *in vivo*. The PAC-3 motifs analyzed are shown in the dashed boxes in Fig 5A. In these experiments, we used the Δ*pac-3* complemented strain and the anti-mCherry antibody. Chromatin was collected from mycelia subjected to alkaline pH stress or not, and the binding of PAC-3 to all gene promoters was analyzed by PCR using the oligonucleotides described in S2 Table. As a positive control of the experiments, the input DNA (I) was analyzed, and as negative controls, a fragment of the ubiquitin gene lacking the PAC-3 motif and the non-immunoprecipitated reactions (N) were used. The DNA fragments amplified in the ChIP-PCR assays (Fig 5B) were quantified by ImageJ and the results are shown in the graphs (Fig 5C). PAC-3 was able to significantly bind to all motifs analyzed both before and after pH stress, with the exception of the motifs in the *pal-2* (the *palB* homolog) and *pac-3* (the *pacC* homolog) promoters, which were bound only at alkaline pH (Fig 5B and 5C). Notably, PAC-3 bound to its own gene promoter suggesting a feedback regulation of PAC-3 under this pH (Fig 5C).

The comparison of the *pal* gene expressions with PAC-3 binding to their promoters allowed us to observe that the promoters of genes whose expression was dependent on PAC-3, such as *pal-1* (the *palA* homolog), *pal-2* (the *palB* homolog), *pal-6* (the *palF* homolog), *pal-8* (the *palH* homolog), and *pal-9* (the *palI* homolog) genes, as shown in Fig 4, were bound *in vivo* by PAC-3 at alkaline pH. Although the expression of the *pal-3* (the *palC* homolog) gene was not influenced by alkaline pH or by PAC-3 (Fig 4), the transcription factor bound to its gene promoter under both conditions (Fig 5C). In this case, PAC-3 was able to bind to the promoter although did not influence its expression. Finally, PAC-3 did not bind to the ubiquitin gene fragment, which has no PAC-3 motif (Fig 5B and 5C) (*P* < 0.01), showing the specificity of the transcription factor binding. All these results show that PAC-3 binds to most of the *pal* promoters *in vivo*, including its own gene promoter, and may influence their expression at normal and alkaline pH.

**Fig 6:**
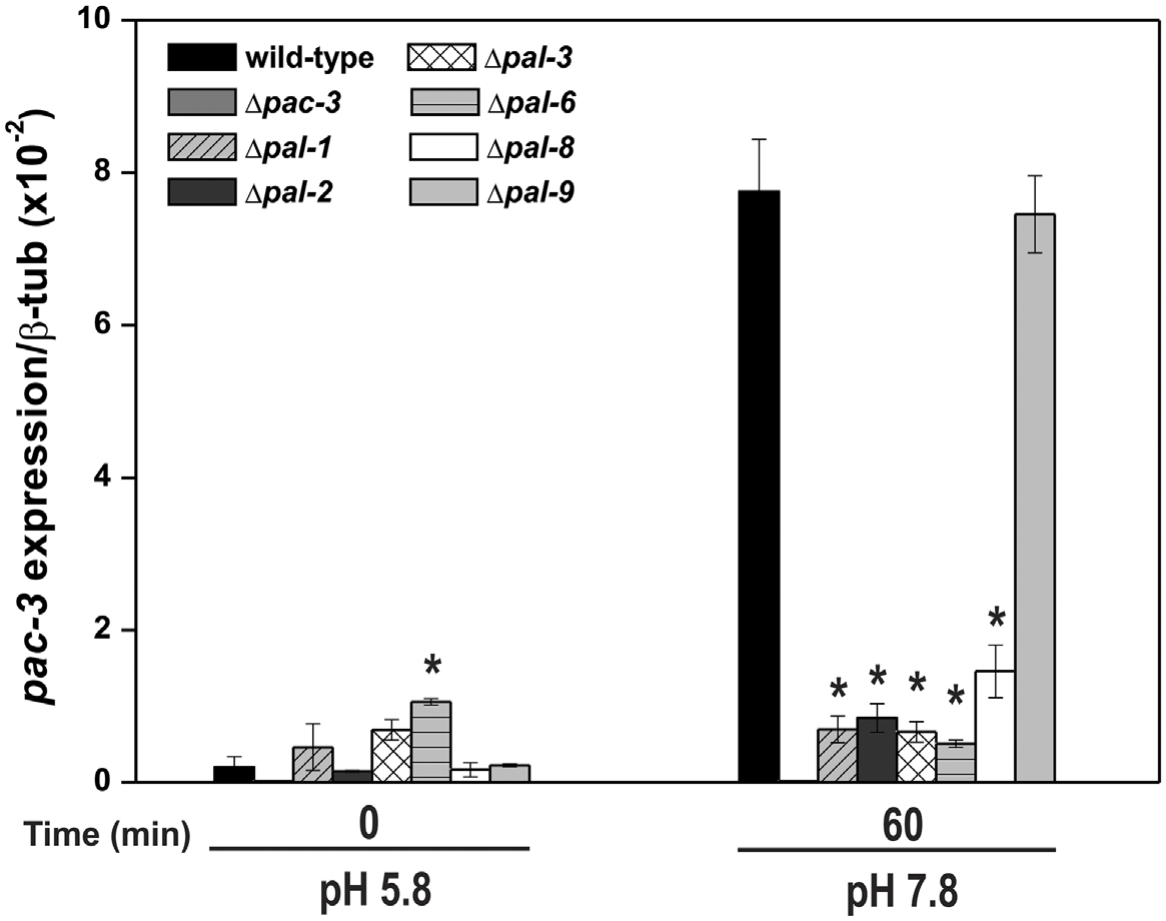
The expression of the *pac-3* gene at normal and alkaline pH. Samples from the wild-type and from all *pal* mutant strains cultured at pH 5.8 for 24 h and then shifted to pH 7.8 for 1 h were used to extract total RNA. Gene expression analysis was performed by RT-qPCR in the StepOnePlus^™^ Real-Time PCR system (Applied Biosystems) using the Power SYBR^®^ Green and specific primers. At least three biological replicates were carried out, and the data were analyzed using the relative quantification standard curve method. Bars indicate the standard deviation from the biological experiments. The *tub-2* gene was used as the reference gene, and the pH 5.8 wild-type was used as the reference sample. *Asterisks indicate significant differences compared to the wild-type at the same pH (Student’s *t*-test, *P* < 0.01).

Since the PAC-3 transcription factor bound to *pac-3* and to the *pal* promoters at alkaline pH, and that *pac-3* was highly expressed in the wild-type strain grown under the same condition, we asked whether the *pac-3* expression could be influenced by the PAL components of the signaling pathway. To investigate this, we analyzed the expression of the *pac-3* gene in mycelia samples from the wild-type and all Δ*pal* strains grown at pH 5.8 for 24 h (control) and in mycelia grown at pH 5.8 and shifted to pH 7.8 for 1 h (Fig 6). We observed very high expression of *pac-3* in the wild-type strain at pH 7.8, in agreement with the results shown in Fig 4. However, very low expression was observed in most of the Δ*pal* mutants at both pH, indicating a negative effect of the PAL components on the *pac-3* expression. These results also suggest that a functional pH signaling pathway is required for proper *pac-3* expression. Thus, we hypothesized that PAC-3 activation in *N*. *crassa* results from proteolytic processing and gene expression regulation by the PAL and PAC-3 components. Interestingly, PAL-9 (the PalI homolog), which did not influence growth at pH 7.8, as shown in Fig 1, also did not influence the *pac-3* expression. However, it was bound by PAC-3 (see Fig 5C) and its expression was influenced by PAC-3 at both pH values.

### PAC-3 shuttles between the nucleus and cytoplasm and may require importin-α

**Fig 7:**
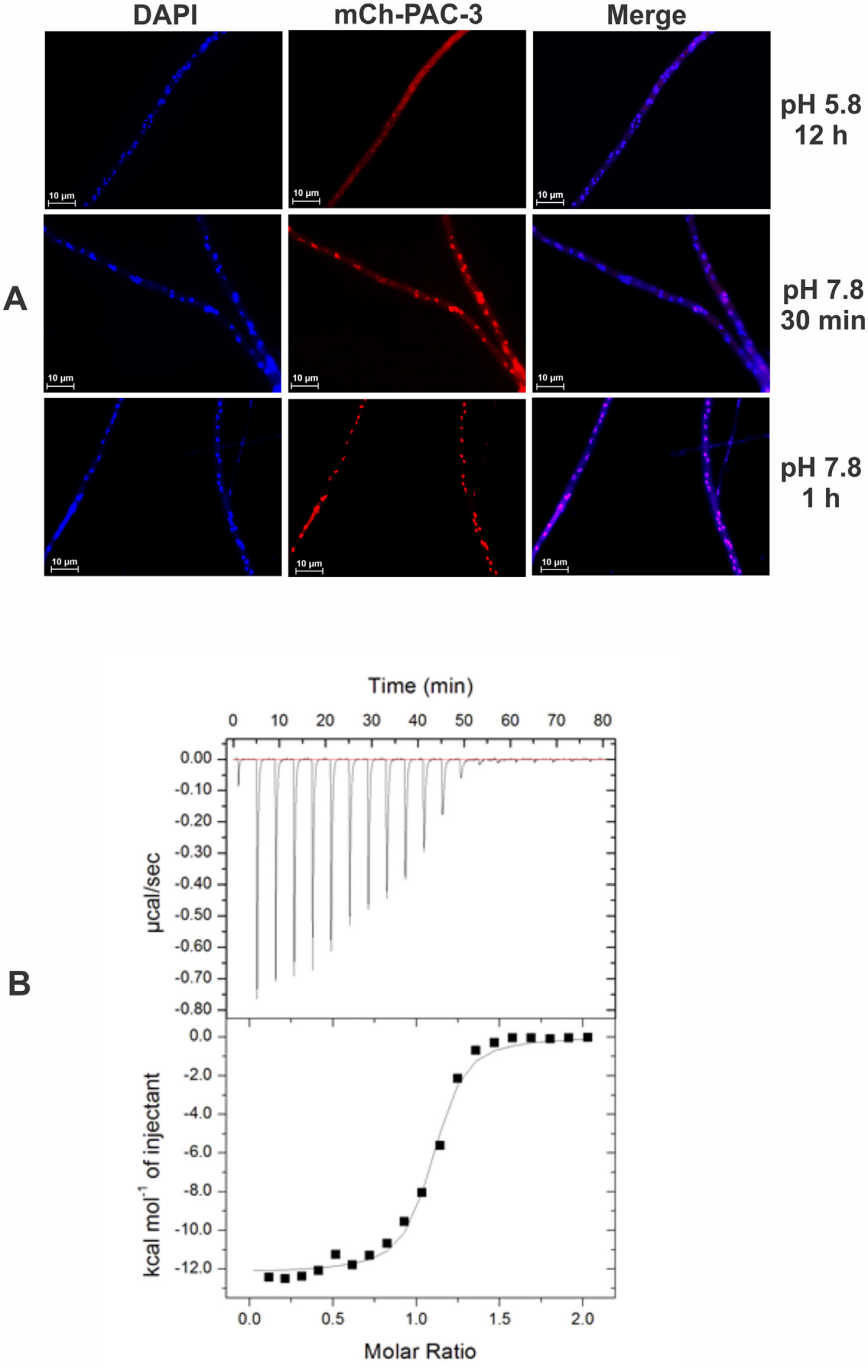
Subcellular localization of PAC-3. **(A)** The PAC-3 protein translocates to the nucleus at alkaline pH. Conidia from the Δ*pac-3* complemented strain were grown onto coverslips in liquid VM medium containing 1% sucrose, pH 5.8 at 30°C for 12 h. After this period, mycelia were transferred to VM medium containing 1% sucrose, pH 7.8 at 30°C (alkaline pH) for 30 min and 1 h. The mycelia were fixed with formaldehyde in PBS, the nuclei were stained with DAPI, and the fluorescence was visualized. Fluorescence was evaluated using the microscope AXIO Imager.A2 (Zeiss) at a magnification of 100 X. The results from one of three independent experiments are shown. **(B)** Isothermal Titration Calorimetry analysis of the PAC-3 NLS peptide binding to importin-α at 20°C. The upper panel corresponds to the thermogram of the replicate titrations (thermal power as a function of time). The lower panel displays the binding isotherm integrated data (kcal mol^-1^ of injectant versus molar ratio of PAC-3 NLS to importin-α).

Since the PAC-3 transcription factor binds to the *pal* and *pac-3* promoters at normal and/or alkaline pH and likely regulates their gene expression under the same conditions, we decided to analyze the subcellular localization of PAC-3 under pH stress. To assess this, we used the Δ*pac-3* complemented strain, in which PAC-3 is produced as an N-terminus mCherry-tagged fusion protein under the control of the *ccg-1* promoter. For this, conidia were germinated at pH 5.8 for 12 h and then transferred to pH 7.8 for 30 min and 1 h. The mCh-PAC-3 was predominantly located in the cytoplasm at pH 5.8; however, after 1 h of transfer to pH 7.8, PAC-3 was detected predominantly in the nuclei (Fig 7A). These data are consistent with the results of PAC-3 binding to the *pal* and *pac-3* promoters and the expression modulation of these genes at alkaline pH, a condition where PAC-3 should be predominantly located in the nucleus. Our results are also in agreement with those described for *A*. *nidulans* by Peñalva et al. [8], who observed a preferential nuclear localization of the PacC^27^ and PacC^53^ forms under the same condition.

Since importin-α, together with importin-β, recognizes cargo proteins that contain nuclear localization sequences (NLS) and translocates into nucleus, we investigated the PAC-3 translocation by analyzing *in vitro* the interaction between importin-α and a peptide corresponding to the putative nuclear localization signal (NLS) of PAC-3. For this assay, recombinant *N*. *crassa* importin-α from *E*. *coli* was produced, and its interaction with the PAC-3 NLS peptide was analyzed by ITC to obtain the dissociation constant and the thermodynamic values for the formation of this complex. ITC data analyses resulted in a single binding site model indicating that one peptide binds to one protein with strong affinity (Fig 7B), with a dissociation constant (K_d_) of 0.39 ± 0.074 μM, enthalpy (ΔH) of -12.10 ± 0.17 kcal mol^-1^ and entropy (ΔS) of -12.20 cal mol^-1^. The negative enthalpy and entropy suggest that the hydrogen bonds play an important role in this interaction and that conformational changes are unfavorable, which are consistent with the interaction between the NLS sequences and importin-α [42] and in other organisms [27]. The data obtained from the titration of the PAC-3 NLS peptide to importin-α showed similar K_d_ values to ITC assays of importin-α and NLS peptides from other analyses [42–44].

## Discussion

All microorganisms need to develop the ability to adapt to environmental pH since it strongly impacts cell growth and development. The Pac/Rim in filamentous fungi and yeast is the best-characterized signaling pathway involved in the pH stress response, and numerous reports have described its importance in different cellular processes. *N*. *crassa* shares all six *A*. *nidulans* Pal orthologs along with the ESCRT proteins required for signal sensing and proteolytic activation of PAC-3 in response to ambient alkaline pH. In this work, we investigated the components of the *N*. *crassa* pH signaling pathway regarding their characterization upon transition from neutral to alkaline pH conditions. All Δ*pal* mutant strains showed reduced growth and low conidiation at normal growth pH, consistent with previously reported results [26]. In addition, the mutant strains showed an inability to grow at alkaline pH, the same phenotype described for the *A*. *nidulans pal* mutants [45]. The Δ*pal-9* strain, however, exhibited a wild-type phenotype. PAL-9 is the *A*. *nidulans* PalI component, which is located in the plasma membrane and together with PalH and PalF establishes the ambient pH signal. Although unnecessary for growth at alkaline pH, *pal-9* expression is regulated by the PAC-3 transcription factor following transition from normal to alkaline pH in *N*. *crassa*. This is quite surprising because in *A*. *nidulans* the PalI component is required for normal growth at pH 8 compared to the wild-type strain under the same condition [45].

An interesting characteristic of the *N*. *crassa* mutant strains, which has not been reported in any of the mutant strains of the pH signaling pathway, is the high production of the pigment melanin, which is important for cell protection in diverse microorganisms and is associated with virulence in many human pathogenic fungi [46, 47]. In *N*. *crassa*, melanin accumulation by most of the mutant strains in the pH signaling pathway could not be attributed to cell protection under adverse conditions, since the pigment accumulation was high at normal growth pH. This result suggests that the components of the signaling pathway, either directly or indirectly, affect the melanin production under normal growth conditions. To identify a regulatory mechanism connecting both processes, pH signaling and melanin accumulation, we demonstrated that the tyrosinase gene was over-expressed in all mutant strains, mainly in the Δ*pac-3* (the *pacC* homolog) strain, but not in Δ*pal-9* (the *palI* homolog) strain. Thus, PAC-3 appears to be the main regulator of tyrosinase expression; it is the final component of the signaling pathway whose activation results from an active signaling cascade. All data are coincident with the high number of PAC-3 motifs in the tyrosinase promoter and with the ability of PAC-3 to bind to the promoter in a pH-independent manner. Melanin is an insoluble compound and is associated with virulence in numerous fungal pathogens, such as *C*. *neoformans* [48] and *Paracoccidioides brasiliensis* [49], among others. In *N*. *crassa*, protoperithecia differentiation was previously correlated with tyrosinase activity and melanin formation [50]. More recently, Park et al. [51] suggested a role for the MAK-1 pathway in melanin production in *N*. *crassa* by modulating the tyrosinase gene expression under nitrogen starvation. Mutant strains in two components of this pathway (Δ*mek-1* and Δ*mak-1*) exhibited high tyrosinase gene expression and accumulated melanin [51]. In addition to the MAK-1 pathway, the pH signaling pathway, among other pathways, was also reported to be required for protoperithecia formation [26], indicating the requirement of different signaling pathways for female development in *N*. *crassa*. The results presented in this work connect all these data by demonstrating that the pH signaling pathway controls tyrosinase gene expression and, as a consequence, the melanin accumulation, which is required for female development. However, the influence of the pH signaling pathway in both processes, protoperithecia formation and melanin production, may be modulated by different regulatory mechanisms because the mutant strains in this pathway are unable to develop protoperithecia but they over-express tyrosinase and accumulate melanin. Additional signaling pathways could play a role in connecting both processes.

We showed here that most of the pH signaling pathway components, but not all, were regulated at the gene expression level by ambient pH, which suggests the existence of feedback regulation on these genes. Such regulation in the pH pathway components was previously reported only for the *C*. *albicans RIM8* gene, which was described as transcriptionally repressed at alkaline pH [19, 52]. In *N*. *crassa*, the expression of the *pal-6* homolog was also negatively regulated by alkaline pH, consistent with the results described in *C*. *albicans*. However, alkaline pH also positively regulates several *pal* genes (*pal-1*, *pal-2*, and *pal-9*) in *N*. *crassa*, suggesting that opposing regulatory mechanisms affect the pH signaling components. Interestingly, while the *pal-9* expression was regulated by alkaline pH, the PAL-9 protein appears to play a minor role in pH signaling transduction in *N*. *crassa* since the Δ*pal-9* mutant strain did not display growth defects at alkaline pH. This is in contrast to what was reported in *S*. *cerevisiae* and *C*. *albicans*, where the Rim9 homolog is necessary for Rim101 cleavage and therefore fully required for pH signaling transduction [17, 53]. The expression of several *pal* genes, including *pal-6*, *pal-8* and *pal-9*, was also dependent on the PAC-3 transcription factor, which indicates that they require an active signaling pathway for normal expression. This result may be a consequence of the ability of PAC-3 to bind to all *pal* promoters at both pH conditions, likely regulating the expression of most *pal* genes.

The *pac-3* expression was also negatively modulated irrespective of both pH by the PAL components in *N*. *crassa*, with the exception of PAL-9, indicating the existence of a cross-regulation among all components of this signaling pathway and a self-regulation on *pac-3* expression. Considering this result, we may suggest that this regulation may be a consequence of the lack of an active PAC-3 in the *pal* mutant strains, which, once activated, could bind to its own promoter at alkaline pH, activating its expression. In *A*. *nidulans*, Trevisan et al. [54] described the existence of alternative RNA splicing of the *palB* gene, depending on the growth conditions, which could affect the PacC protein activity. We also cannot preclude the existence of additional proteins involved in such cross-regulation, similar to what was recently described for the PacX protein in *A*. *nidulans* [21], which was identified to play a role in *pacC* gene repression.

**Fig 8:**
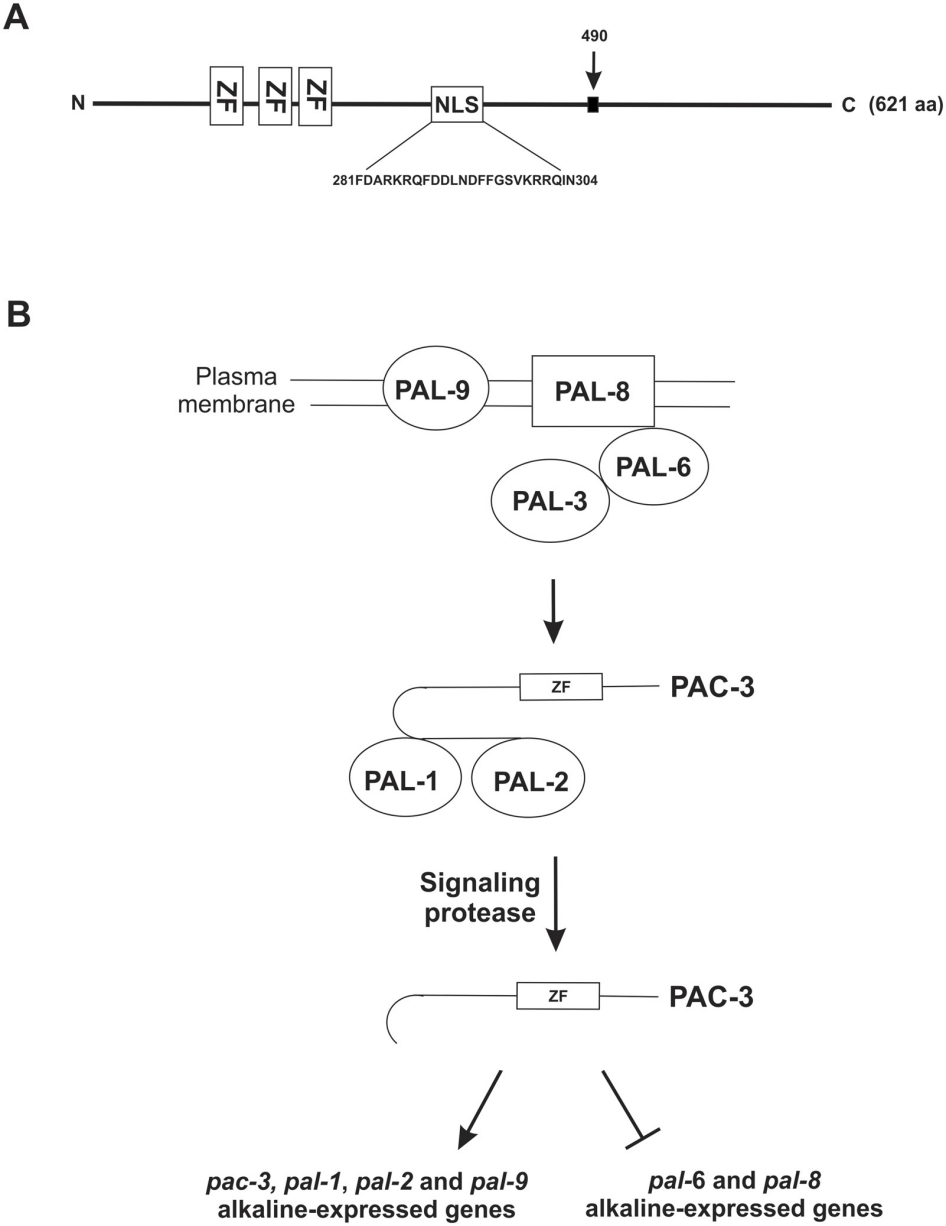
Proposed model for the alkaline pH signaling in *N*. *crassa*. **(A)** The PAC-3 protein contains 621 amino acid residues and has three C_2_H_2_ zinc-finger domains encompassing the amino acid from 95 to 183. The NLS sequence is shown between amino acid residues 281 and 304. The black arrow indicates the putative protease-processing site at amino acid 490. **(B)** *N*. *crassa* has the six *A*. *nidulans* Pal homologues. External pH signaling may involve the PAL-8, PAL-9, PAL-6 and PAL-3 complex. PAL-1 may act together with PAL-2 and may recruit the PAC-3 protein. PAC-3 undergoes only one proteolytic processing and is likely involved in the activation of the *pal-1*, *pal-2*, *pal-9*, and *pac-3* genes and the repression of the *pal-6* and *pal-8* genes at alkaline conditions. aa, amino acid; ZF, C_2_H_2_ zinc finger; NLS, nuclear localization signal.

We demonstrated here that PAC-3 may be processed in a single proteolytic step, similar to what was described for *S*. *cerevisiae* [17] and *C*. *albicans* [18]. In addition, as in *C*. *albicans*, PAC-3 processing was also observed at acidic pH, which could explain the localization of PAC-3 to the nucleus at normal pH growth conditions and its regulatory function at the same pH. The results presented here regarding the protein processing at acidic pH confirm previous results obtained by our group [23] using different experimental approaches. However, we previously were unable to detect the unprocessed PAC-3 [23]. We also showed here that PAC-3 translocates to the nucleus at alkaline pH and that this process may occur by the classical nuclear import pathway, in which importin-α binds to a specific NLS, known as classical NLSs. ITC assays between PAC-3 NLS and importin-α demonstrated that these molecules have an affinity compatible with the interaction between a classical NLS peptide and importin-α [42]. A classical bipartite NLS presents two basic clusters that binds to different regions of the importin-α [38] with a stoichiometry of 1:1, and its consensus sequence is generally accepted as **KR**X_10-12_**K**(K/R)X(K/R), where X corresponds to any residue, and residues in bold indicate critical residues [55]. PAC-3 NLS displays all these critical requirements, except for the last position (K/R) (Fig 8A), for its sequence to be classified as a classical bipartite NLS. ITC results also supported this identification because a stoichiometry of 1:1 for the NLS peptide/importin-α complex is characteristic of a bipartite NLS, while most classical monopartite NLSs bind with a stoichiometry of 1:2 (protein:peptide). Thus, the data presented here indicate that this NLS region is responsible for the recognition of the PAC-3 transcription factor by importin-α, and these components may form a complex that permits PAC-3 to be translocated to the nucleus under specific conditions. The *N*. *crassa* importin-α used in our work was first identified by Foss et al. [56] as important for DNA methylation, and later described as required for heterochromatic formation and DNA methylation [57, 58].

Finally, we may conclude that the *N*. *crassa* PAC-3 transcription factor cycles between the cytoplasm and nucleus under pH alkaline stress, either up- or down-regulating the expression of pH-responsive genes. Based on our findings, we propose a model of the pH signaling pathway in *N*. *crassa* (Fig 8B). According to the results, the pathway requires the same Pal/Rim components described in *A*. *nidulans* and *S*. *cerevisiae*, sharing characteristics with both organisms. We suggest that the PAC-3 transcription factor, once activated, regulates the *pal* gene expression and that this regulation may be directly mediated by PAC-3 because it binds to all *pal* promoters.

## Supporting Information

S1 Fig Morphological analysis of the Δ*pac-3* complemented strain. **(A)** Growth of the wild-type, Δ*pac-3* and Δ*pac-3* complemented (Δ*pac-3 pac-3*^+^) strains in tubes containing solid VM medium plus 2% sucrose at pH 5.8. **(B)** Growth of the same strains in Petri dishes containing solid VM medium plus 2% sucrose at pH 5.8 and 7.8 for 24 h.
(TIF)

S1 Table Protein family or domain classification, annotation, and biochemical and structural characteristics of the proteins. (DOC)

S2 Table Oligonucleotides used in this study. (DOC)
