# Supplementary figures and images for: Molecular Components of the Neurospora crassa pH Signaling Pathway and Their Regulation by pH and the PAC-3 Transcription Factor
Source: PLoS One. 2016 Aug 24;11(8):e0161659. doi: 10.1371/journal.pone.0161659 (PMC4996508; doi:10.1371/journal.pone.0161659)

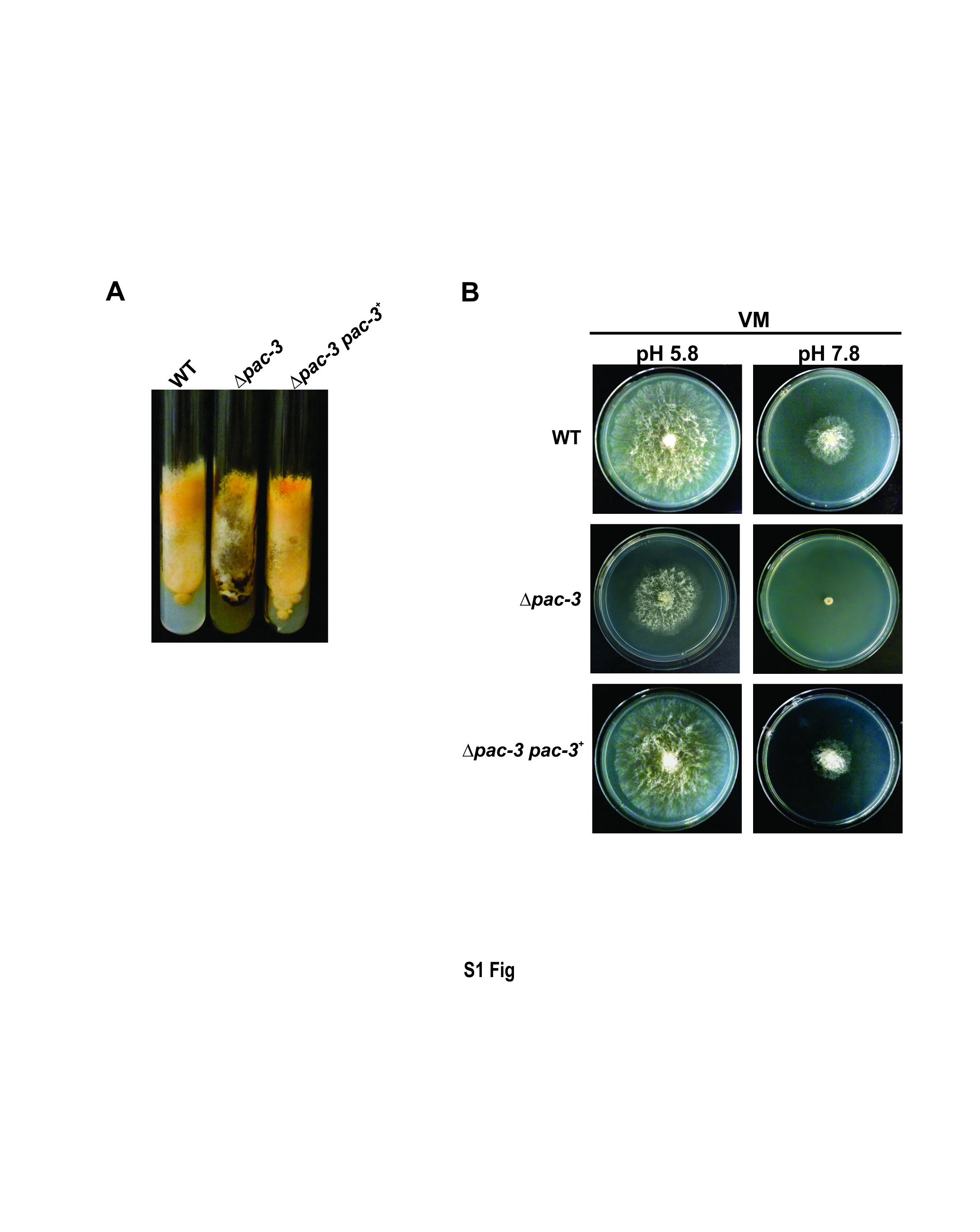

Supplement: S1 Fig — (A) Growth of the wild-type, Δpac-3 and Δpac-3 complemented (Δpac-3 pac-3+) strains in tubes containing solid VM medium plus 2% sucrose at pH 5.8. (B) Growth of the same strains in Petri dishes containing solid VM medium plus 2% sucrose at pH 5.8 and 7.8 for 24 h. (TIF) [file pone.0161659.s001.tif]
