# Supplementary material for: Molecular Components of the Neurospora crassa pH Signaling Pathway and Their Regulation by pH and the PAC-3 Transcription Factor
Source: PLoS One. 2016 Aug 24;11(8):e0161659. doi: 10.1371/journal.pone.0161659 (PMC4996508; doi:10.1371/journal.pone.0161659)
Supplement: S1 Table — (DOC) [file pone.0161659.s002.doc]

**S1 Table. Protein family or domain classification, annotation, biochemistry and structural characteristics of the proteins^a^**.

| **FGSC#** | **Mating type** | **ORF** | **Protein family or domain** | **Theoretical MW/pI** | **Annotation of orthologs** | **Gene names^b^** | **Gene names^c^** |
| --- | --- | --- | --- | --- | --- | --- | --- |
| 21931 | a | NCU05876 | BRO-1 and ALIX V | 96.9/6.29 | pH-response regulator protein PalA/Rim20 | *prr-1* | *pal-1* |
| 15867 | A | NCU00317 | Peptidase C2 and Calpain III | 101.46/5.93 | calpain signaling protease PalB/Rim13 | *cpr-8* | *pal-2* |
| 16419 | a | NCU03316 | BRO-1 | 53.45/6.77 | pH-response regulator protein PalC/YGR122W | *prr-2* | *pal-3* |
| 22412 | a | NCU03021 | Arrestin | 102.83/5.69 | pH-response regulator protein PalF/Rim8 | *prr-3* | *pal-6* |
| 16099 | a | NCU00007 | PalH | 83.99/9.09 | pH-response regulator protein PalH/Rim21 | *prr-4* | *pal-8* |
| 13378 | a | NCU01996 | SUR7/PalI | 73.56/8.99 | pH-response regulator protein PalI/Rim9 | *prr-5* | *pal-9* |
|  | a | NCU00090 | C_2_H_2_ zinc finger | 67.3/7.19 | pH-response transcription factor PacC/Rim101 | *pacc-1* | *pac-3***^d^** |

^a^ The identification of each strain was made according to the Fungal Genetics Stock Center (www.fgsc.net) number. Theoretical estimative of physical and chemical characteristics was performed according to ProtPAram tools (www.expasy.org/tools/protpar-ref.html). The protein family or domains were classified according to Pfam 28.0 (pfam.sanger.ac.uk). MW, molecular weight; pI, isoeletric point. Annotation of ortholog proteins in *Aspergillus nidulans* and *Saccharomyces cerevisiae*, respectively.

**^b^** Nomenclature proposed by Alan Radford (http://www.bioinf.leeds.ac.uk/~gen6ar/newgenelist/genes/browse.html).

**^c^** Nomenclature proposed in this work based on the nomenclature proposed by Perkins et al*.* [39].

**^d^** The construction of the knockout strain was described in Cupertino et al. [23]. The gene was named as *pacC;* we renamed here as *pac-3* gene according to the *Neurospora crassa* nomenclature [39].
