# Supplementary material for: Molecular Components of the Neurospora crassa pH Signaling Pathway and Their Regulation by pH and the PAC-3 Transcription Factor
Source: PLoS One. 2016 Aug 24;11(8):e0161659. doi: 10.1371/journal.pone.0161659 (PMC4996508; doi:10.1371/journal.pone.0161659)
Supplement: S2 Table — (DOC) [file pone.0161659.s003.doc]

**S2 Table**. Oligonucleotides used in this study.

| **Primer** | | **Sequence**^a^ **(5’→3’)** | | **Source** | **Name** | **Position^b^** |  |
| --- | --- | --- | --- | --- | --- | --- | --- |
| Δ*pac-3 complementation* | | | | | | |  |
| N-mChPACC-F | GCACTAGT**GGAGGAGGAGGAGGAGGA**ATGTCGTCCACACCAGCCC | | NCU00090 | | - | +1 to +19 |  |
| N-mChPACC-R | CTAGTCTAGATTAGTTGATGCGAGGAAGAAC | | NCU00090 | | - | +2021 to +2041 |  |
| *Real-time PCR* | | |  | |  |  |  |
| tyrosinase_F | CGACGAGTATAATCTGGAGGA | | NCU00776 | | - | +2044 to +2064 |  |
| tyrosinase_R | CTGGCGAGAGTAATGTGG | | NCU00776 | | - | +2126 to +2143 |  |
| qPac3-F | CAAGCATCGACCCGTATCAT | | NCU00090 | | - | +1377 to +1396 |  |
| qPac3-R | TGGTGAGTGACCCGAAGTA | | NCU00090 | | - | +1486 to +1504 |  |
| qPal1-F | CTGGATGCGGCCTATTACAA | | NCU05876 | | - | +2065 to +2084 |  |
| qPal1-R | CCCTCCACTGTTCTACAATCTG | | NCU05876 | | - | +2143 to +2164 |  |
| qPal2-F | GGGATTGGAGGTGGACATATAC | | NCU00317 | | - | +2719 to +2740 |  |
| qPal2-R | CTCCCTCGTTCTTAAACCTAGAC | | NCU00317 | | - | +2804 to +2826 |  |
| qPal3-F | GGCGATTGCTTGGTTGAATG | | NCU03316 | | - | +981 to +1000 |  |
| qPal3-R | AACCTCCGCTCCTCTTACT | | NCU03316 | | - | +1048 to +1066 |  |
| qPal6-F | GAAGATTATCACGGTGGTGGAT | | NCU03021 | | - | +2639 to +2660 |  |
| qPal6-R | GACCCAGGGCGATGATTATT | | NCU03021 | | - | +2744 to +2763 |  |
| qPal8-F | ATGGCACGCACTTACACCAACA | | NCU00007 | | - | +1725 to +1746 |  |
| qPal8-R | TTGCTGCGAACTCCTCAACT | | NCU00007 | | - | +1836 to +1855 |  |
| qPal9-F | AAGAAGGGCCCAACGACGTTTA | | NCU01996 | | - | +1696 to +1717 |  |
| qPal9-R | AATCACTGGCCACTGTAGCTGT | | NCU01996 | | - | +1812 to +1833 |  |
| 4054Tub-F | CCTCCACCTTCGTCGGTAACTCC | | NCU04054 | | - | +1669 to +1691 |  |
| 4054Tub-R | GGTACTGCTGGTACTCGGAGACG | | NCU04054 | | - | +1832 to +1854 |  |
| *ChIP-PCR* | | | | |  |  |  |
| pal1p-F | GCATTCGTACCTCTACCCACCG | | p*pal-1* | | *pal-1* (129 bp) | -247 to -226 |  |
| pal1p-R | TGACATCAATCAACCACCAGCCC | | p*pal-1* | | *pal-1* (129 bp) | -141 to -119 |  |
| pal2p-F | CCAACGACGTTTACGTGCCGCC | | p*pal-2* | | *pal-2* (162 bp) | -794 to -775 |  |
| pal2p-R | CAGTGTCGGTGGAGATTCTGGC | | p*pal-2* | | *pal-2* (162 bp) | -654 to -633 |  |
| pal3p-F | GAGGAGGATAGTCGAGACCCCAC | | p*pal-3* | | *pal-3* (119 bp) | -963 to -941 |  |
| pal3p-R | ACCACCTCTGTTTGGCGACTAC | | p*pal-3* | | *pal-3* (119 bp) | -866 to -845 |  |
| pal6p-F | CTTCCCTTTCTCCTCCACCGTC | | p*pal-6* | | *pal-6* (121 bp) | -691 to -670 |  |
| pal6p-R | TGGACAAGCAAGGCCTGGA | | p*pal-6* | | *pal-6* (121 bp) | -589 to -571 |  |
| pal8p-F | GTTCCCTCGACCACTTCCCA | | p*pal-8* | | *pal-8* (135 bp) | -662 to -643 |  |
| pal8p-R | GGGAATATTGCGCCGCGG | | p*pal-8* | | *pal-8* (135 bp) | -545 to -528 |  |
| pal9p-F | CTCCTCCATCTGCCCTTTCCAA | | p*pal-9* | | *pal-9* (159 bp) | -914 to -893 |  |
| pal9p-R | CCAAGATTACAGCCGGGAGTGG | | p*pal-9* | | *pal-9* (159 bp) | -777 to -756 |  |
| pac3p-F | GCTGCCAAGTCTTTTCGCCAG | | p*pac-3* | | *pac-3* (196 bp) | -617 to -597 |  |
| pac3p-R | CGCGCGCAGGAAGAATCG | | p*pac-3* | | *pac-3* (196 bp) | -439 to -422 |  |
| tyrp-F | TTTAGCGCCCGGTGTCCA | | ptyrosinase | | tyrosinase (111 bp) | -1852 to -1835 |  |
| tyrp-R | ACAAGTAGTCCCGATCCGTGG | | ptyrosinase | | tyrosinase (111 bp) | -1762 to -1742 |  |
| qUbi-F | CGAGTCTTCGGATACGATTG | | *ubiquitin* gene | | *ubiquitin* (108 bp) | +805 to +824 |  |
| qUbi-R | CCATCCTCCAACTGCTTAC | | *ubiquitin* gene | | *ubiquitin* (108 bp) | +894 to +912 |  |

^a^ The *Spe*I and *Xba*I restriction sites are underlined in the N-mChPACC-F and N-mChPACC-R sequences, respectively. The nucleotides in bold in the oligonucleotide N-mChPACC-F represent the nucleotide sequence encoding for 6-Gly.

^b^ The DNA oligonucleotides are positioned according to the gene ATG start site from genomic DNA.
